# Practice patterns of paediatric surgeons on treating pilonidal sinus disease — a national survey study

**DOI:** 10.1007/s00384-025-04959-x

**Published:** 2025-07-28

**Authors:** Giada Corturillo, Marko Jovanovic, Stephan Rohleder, Andreas C. Heydweiller, Oliver J. Muensterer, Dietrich Doll, Christina Oetzmann von Sochaczewski

**Affiliations:** 1 Sektion Kinderchirurgie der Chirurgischen Klinik, Universitätsklinikum Bonn, Bonn, Germany; 2 Institut für Digitale Medizin, Universitätsklinikum Bonn, Venusberg-Campus 1, 53127 Bonn, Germany; 3 Klinik und Poliklinik für Kinderchirurgie, Universitätsmedizin Mainz, Mainz, Germany; 4 Kinderchirurgische Klinik, St. Marien Hospital Bonn, Bonn, Germany; 5 Kinderchirurgische Klinik und Poliklinik, Klinikum der Ludwig-Maximilians-Universität München, Dr. von Haunersches Kinderspital, Munich, Germany; 6 Klinik für Proktochirurgie und Pilonidalsinus, St. Marienhospital Vechta, Vechta, Germany

**Keywords:** Pilonidal sinus disease, Child, Survey, Paediatric surgery, Germany

## Abstract

**Purpose:**

Paediatric pilonidal sinus disease is considered a separate entity of disease due to differing recurrence dynamics. However, there are almost no data on real-world surgical care and practice patterns for children and adolescents. We therefore aimed to gather such data surveying a representative sample of German paediatric surgeons.

**Methods:**

Some 101 German paediatric surgical departments and surgeries with inpatient beds were surveyed for their surgical approach to paediatric pilonidal sinus disease. The survey included demographics, information on practice setting, as well as the primary and secondary approach to pilonidal disease and three virtual patient scenarios.

**Results:**

A total of 40 institutions (33 departments and 7 office-based paediatric surgeons) responded (recall rate 40%). Of these, 18/40 reported performing 10–20, and 9/40 perform 5–10 pilonidal sinus operations annually. 17/39 respondents have less than 10% recurrences among their patient cohort and 15/39 operate on 11–20% recurrences among their patients. The most frequently reported surgical approach was excision and secondary closure with 17/39, followed by excision and vacuum-assisted closure (13/39), and pit-picking (12/39). Intraoperative use of blue dyes report 15/39 and 29/39 continue postoperative outpatient care at their institution. Acute pilonidal sinus with abscess is treated with a two-staged approach within four weeks by 15/39 while 11 institutions wait more than four weeks until definitive surgery. In recurrent cases, the majority of 20/39 does not switch their approach, while 13/39 switch to excision and secondary closure, and 11/39 switch to excision and vacuum-assisted closure.

**Conclusion:**

German paediatric surgeons prefer traditional approaches to pilonidal sinus diseases, but pit-picking is frequently used. Neither the adult-based national guideline recommendations nor paediatric treatment algorithms have been widely implemented. The reasons for these deviations from recommendations and favouring traditional approaches remain unclear.

**Supplementary Information:**

The online version contains supplementary material available at 10.1007/s00384-025-04959-x.

## Introduction

The incidence of pilonidal sinus disease is increasing [1, 2], including children and adolescents [3, 4]. Despite this development, paediatric pilonidal sinus disease has long been a blind spot [5, 6], which has changed only recently. Paediatric pilonidal sinus disease has been suggested to be a different entity among the spectrum of pilonidal sinus disease. This has been based on its specific temporal peculiarities in recurrence dynamics [6–9], although the causative factors for these differences remain elusive. It resulted in several, slightly differing, suggested treatment algorithms focusing on repeat minimally invasive procedures [10–12]. These algorithms aimed to address the issue of increased recurrences that occur earlier than in adults in order to reduce the impact on quality of life and development of children. It is, however, unknown whether these recommendations have been implemented into daily practice. In adults, the surgical community has gained insights into treatment approaches from several countries: Some preferring traditional excision and open wound treatment, such as in Germany [13], Russia [14], and the Netherlands [15]. On the contrary, Norwegian [16] and Australian [17] surgeons favoured flap closure, as do Austrian [18] and, to a lesser extent, Swiss [18] surgeons. The latter frequently use minimally invasive techniques, as do Danish surgeons, although excisional procedures were quite common there [19]. British surgeons, on the other hand, used excision and open wound treatment commonly, too [20]. For paediatric surgeons, only one assessment from Turkey is available in which the vast majority of respondents selected excision and primary midline closure, followed by excision and secondary healing [21]. In order to provide additional insight into treatment reality of children and adolescents with pilonidal sinus disease, we surveyed German paediatric surgical departments and paediatric surgical practices with inpatient beds for their approach towards paediatric pilonidal sinus disease. Surveying German paediatric surgeons felt reasonable, because Germany is a country whose paediatric surgical care is highly decentralised [22]. This is in sharp contrast to other countries, such as the Netherlands or Scandinavian countries, which have a highly centralised paediatric surgical care [23, 24]. We aimed to assess management strategies and adherence to guidelines and treatment recommendations for paediatric pilonidal sinus disease among German paediatric surgeons.

## Methods

A total of 101 departments, including 90 paediatric hospitals and 11 office-based practices with associated inpatient beds, were identified through the list of all German paediatric and adolescent surgical departments. This list is accessible through the website of the *Deutsche Gesellschaft für Kinder- und Jugendchirurgie* (German Association for Paediatric and Adolescent Surgery). Participation in the survey was limited to the identified institutions on the institutional level. Using the websites of the listed paediatric surgical doctor’s surgeries, we excluded all practices without inpatient beds from study participation. The email addresses or phone numbers of the respective head of department or practice, which could be found on their respective public websites, were collected. The latter were only used in case there was no email contact provided in order to obtain an email address to send the invitation to.

The survey consisted of 14 questions regarding the treatment of acute and asymptomatic pilonidal sinus disease in the paediatric and adolescent population in Germany. We asked for the annual number of operations, the relative share of recurrences in the operated numbers, the level of training of the operating surgeons, their standard operative techniques, techniques used in case of recurrence with regard to a change of technique compared to a primary operation, the use of intraoperative dyes, and postoperative treatment. A multiple choice option was allowed in questions asking for the level of training, the standard operative techniques, and the treatment of an asymptomatic pilonidal sinus disease. In addition, three clinical scenarios were presented as typical examples based on the treatment of acute pilonidal sinus with abscess and the treatment of asymptomatic pilonidal sinus: Each case consisted of specific modifications to evaluate how therapy decisions are made for patients of different age groups. A final question refers to the perceived prevalence of pilonidal sinus in the paediatric surgical community. Both the translated survey questionnaire (Supplementary Information 1) and the original survey questionnaire in German (Supplementary Information 2) are provided along the manuscript. The *Deutsche Gesellschaft für Kinder- und Jugendchirurgie* (German Association for Paediatric and Adolescent Surgery) fully supported the project promoting its conduction and encouraged members to participate in the survey.

The survey was promoted on the website of the *Deutsche Gesellschaft für Kinder- und Jugendchirurgie* (German Association for Paediatric and Adolescent Surgery) in December 2023. At the same time, eligible institutions were contacted via email, which included a link with an institution-specific code to access the survey via software provided by our institution, which had also been the controller of the data. The use of the institution-related code allowed the identification of institutions which had not yet completed the survey until February 2024, so that a reminder-email could be sent to the selected institutions that same month. The survey did only open after the respondent explicitly stated consent to participate for the surveyed institution in our study.

The software only allowed identifying which institutions had completed the survey, but not their answers to the questions. They were recorded strictly anonymous. Results from the survey were only available for analysis in aggregate form. This adherence to privacy by design ensured that no patient or any other identifiable information would be gathered or stored. Therefore, our study was exempt from ethical approval by law. The General Data Protection Regulation (2016/679/EU) is only applicable to personal data (article 2 paragraph 1), which is defined as any information relating to a natural person (article 4 paragraph 1). By limiting participation to institutions, we ensured that no personal data was collected, as institutions are not natural persons but legal entities. Another approach to avoid processing any personal data was to use ranges instead of absolute numbers when asking for recurrence rates and caseload. This ensured that responding institutions could not be de-anonymised using their publicly available data on caseloads from the quality control reports (*Strukturierter Qualitätsbericht*).

The survey was not formally piloted, but among two senior consultants of two participating academic and a non-academic units, it was informally evaluated for a similar interpretation of the survey items. Likewise, the comprehensibility of the survey items was evaluated. All survey items were reconciled with a member of the German national guideline team.

Inferential statistical analyses were not conducted. Results are presented in a descriptive fashion only.

## Results

The survey was sent to a total of 101 German paediatric surgical units, of whom 40 (40% recall rate) — 33 departments and seven office-based practices with inpatient beds participated. Of the 101 eligible institutions, 25 (seven surgeries and 18 departments) completed the survey between December 2023 and February 2024 after receiving the first email. An additional 15 responses (departments only) came after the reminder-email, between February and August 2024.

**Fig. 1:**
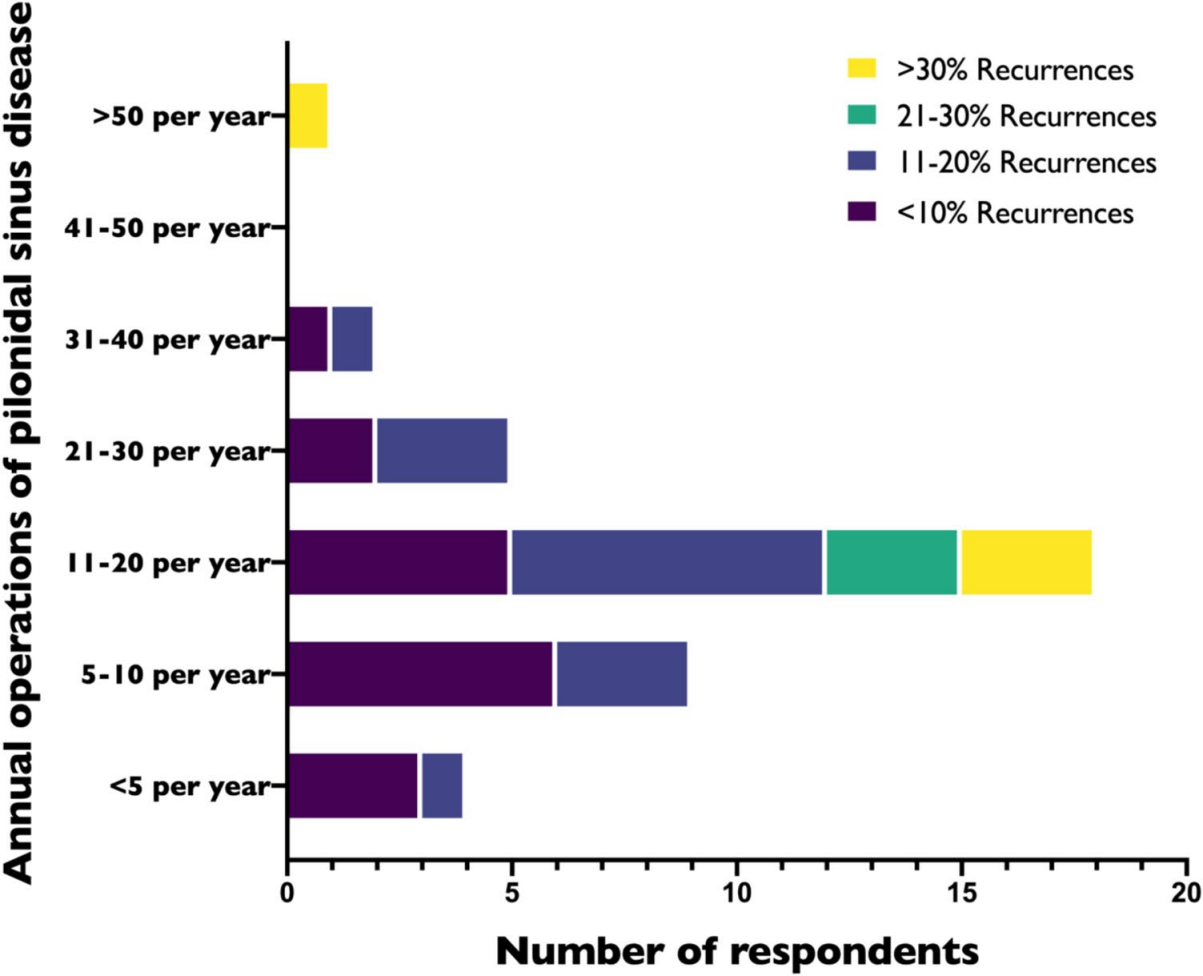
Annual incidence of operations for pilonidal sinus disease in children and adolescents and share of recurrences among paediatric surgical practitioners in Germany

Only one of the participating surgeries stated that they did not perform pilonidal sinus surgery, leaving 39 institutions which answered the remaining questions. Most respondents (18/40; 45%) perform between 11 and 20 pilonidal sinus surgeries per year. Nine institutions (23%) perform between five and ten operations, five institutions (13%) between 21 and 30 operations, and four departments (10%) reported to perform fewer than five annual cases for pilonidal sinus disease. Two respondents reported operating between 31 and 40 pilonidal sinus cases per year, while only one department treated more than 50 annual cases (Fig. 1).

Concerning recurrences among the treated patients, 17 out of 39 institutions (44%) report less than 10% of their cases to be recurrences, while in 15/39 (39%) departments, the percentage of recurrent cases was between 11 and 20% (Fig. 1). A smaller portion of institutions describe a higher percentage of their surgical volume to be conducted on recurrent cases: Three departments (8%) perform 21–30% of surgeries for recurrent disease, while four departments (10%) report more than 30% of cases to be conducted for recurrences of a pilonidal sinus (Fig. 1)(Table 1).

**Table 1:** Recurrence rates stratified by surgical volume

Caseload	RR < 10%	RR 11–20%	RR 21–30%	RR > 30%
< 5 per year	4/39 (10%)	1/39 (3%)	0/39 (0%)	0/39 (0%)
5–10 per year	5/39 (13%)	3/39 (8%)	0/39 (0%)	0/39 (0%)
11–20 per year	5/39 (13%)	7/39 (18%)	3/39 (8%)	3/39 (8%)
21–30 per year	2/39 (5%)	3/39 (8%)	0/39 (0%)	0/39 (0%)
31–40 per year	1/39 (3%)	1/39 (3%)	0/39 (0%)	0/39 (0%)
41–50 per year	0/39 (0%)	0/39 (0%)	0/39 (0%)	0/39 (0%)
> 50 per year	0/39 (0%)	0/39 (0%)	0/39 (0%)	1/39 (3%)

The most frequent surgical approaches to symptomatic pilonidal sinus disease was excision and secondary wound closure (17/39, 44%), followed by excision and vacuum-assisted wound closure (13/39, 33%), and pit-picking (12/39, 31%). Other approaches were less frequently used (Table 2). In recurrent cases, the majority of respondents (20/39, 51%) did not switch their approach and those that did favoured excision and secondary wound healing (13/39, 33%) or excision and vacuum-assisted wound closure (12/39, 31%), while other approaches were seldom used: For recurrent disease, Gips procedure, endoscopic therapy, laser therapy, and marsupialisation were not reported (Table 2).

**Table 2:** Surgical approaches for primary and recurrent pilonidal sinus disease

Procedure	Primary approach [*n*] (%)	Recurrence approach [*n*] (%)
No change in surgical approach	Not applicable	20/39 (51%)
Excision and open wound treatment	17/39 (44%)	13/39 (33%)
Excision and vacuum-assisted closure	13/39 (33%)	12/39 (31%)
Pit-picking	12/39 (31%)	1/39 (3%)
Excision and midline closure	7/39 (18%)	1/39 (3%)
Excision and off-midline closure	6/39 (15%)	2/39 (5%)
Karydakis or Bascom flaps	6/39 (15%)	3/39 (8%)
Limberg or Dufourmentel flaps	5/39 (13%)	3/39 (8%)
Minimally invasive sinusectomy	3/39 (8%)	1/39 (3%)
Endoscopic pilonidal sinus therapy	3/39 (8%)	0/39 (0%)
Gips-procedure (trephination)	1/39 (3%)	0/39 (0%)
Sinus laser ablation of the cyst	1/39 (3%)	0/39 (0%)
Marsupialisation	1/39 (3%)	0/39 (0%)

Our results reveal that most operations were performed either by a consultant (*Facharzt*) (22/39, 56%), by a senior consultant (*Oberarzt*) (21/39, 54%) or by specialty registrars (17/39, 44%). In seven (18%) departments, the head of department also operated on patients with pilonidal sinus disease.

With regard to use of intraoperative dyes, 24 (62%) departments state that they don’t use any, leaving 38% (15/39) of departments using methylene blue, toluidine blue or patent blue.

In most of the cases (29/39, 74%), the postoperative care of the patients remained in the hands of the surgical department after discharge. A surgeons’ practice continued the outpatient care in seven (18%) respondents, and paediatrician or family medicine surgery followed up the cases for the remaining three respondents (8%).

Of the 39 departments participating in the survey, 15 (39%) treated an acute pilonidal sinus with abscess employing a two-staged approach, consisting of abscess drainage followed by definitive surgery within 4 weeks, while 11 institutions (28%) preferred to wait more than four weeks until definitive surgery is performed. The remainder of 13 respondents (33%) preferred a one-stage approach, combining both abscess drainage and definitive procedure in a single operation.

In order to gain additional insight in the current management of typical cases, we asked the institutions how they would manage three distinct cases. In the case of definitive surgical treatment for a 12-year-old girl presenting with an acute pilonidal sinus 6 weeks after abscess drainage, the most commonly selected procedure was excision with secondary wound healing, which was chosen by 13 (33%) institutions, followed by the minimally invasive pit-picking (11/39, 28%). Of the remaining institutions, eight (21%) opted for excision and off-midline primary closure and five (13%) for primary closure in the midline. Only two out of 39 (5%) chose flap surgery. Substantial differences with regard to caseloads of participating institutions could not be identified (Table 3).

**Table 3:** Approach to a 12-year-old girl with an acute abscess for delayed definitive surgery

Caseload	Excision and primary midline closure	Excision and off-midline closure	Excision and open wound treatment	Minimally-invasive approach	Excision and flap closure
< 5 per year	0 (0%)	2/7 (29%)	2/7 (29%)	3/7 (43%)	0 (0%)
5–10 per year	0 (0%)	1/7 (14%)	2/7 (29%)	4/7 (57%)	0 (0%)
11–20 per year	3/18 (17%)	5/18 (28%)	7/18 (39%)	2/18 (11%)	1/18 (6%)
21–30 per year	0 (0%)	0 (0%)	2/5 (40%)	2/5 (40%)	1/5 (20%)
31–40 per year	1/1 (100%)	0 (0%)	0 (0%)	0 (0%)	0 (0%)
41–50 per year	0 (0%)	0 (0%)	0 (0%)	0 (0%)	0 (0%)
> 50 per year	1/1 (100%)	0 (0%)	0 (0%)	0 (0%)	0 (0%)

In another scenario involving the surgical treatment of a 17-year-old boy presenting with an acute pilonidal sinus with abscess formation, most institutions (14/39, 36%) chose excision and secondary wound healing, while 11 (28%) selected minimally-invasive approaches. Among the other options, flap surgery was selected seven (18%) times, primary closure in the midline four times (10%), and off-midline primary closure three times (8%). Substantial differences with regard to caseloads of participating institutions could not be identified (Table 4). When asked whether the choice of procedure would be influenced by the fact that the patient would be staying for at least a year in a low-to-middle income country, 29 out of 39 (74%) departments indicated that this would not affect their treatment decision. Of the 10 departments who would change their decision, the majority (5, 50%) would choose excision and secondary wound healing, three respondents opted for primary off-midline closure, one for primary closure in the midline, and one preferred flap surgery.

**Table 4:** Approach to a 17-year-old boy with an acute abscess for delayed definitive surgery

Caseload	Excision and primary midline closure	Excision and off-midline closure	Excision and open wound treatment	Minimally-invasive approach	Excision and flap closure
< 5 per year	0 (0%)	1/7 (14%)	1/7 (14%)	4/7 (57%)	1/7 (14%)
5–10 per year	1/7 (14%)	0 (0%)	2/7 (29%)	3/7 (43%)	1/7 (14%)
11–20 per year	2/18 (11%)	2/18 (11%)	8/18 (44%)	2/18 (11%)	4/18 (22%)
21–30 per year	0 (0%)	0 (0%)	2/5 (40%)	2/5 (40%)	1/5 (20%)
31–40 per year	1/1 (100%)	0 (0%)	0 (0%)	0 (0%)	0 (0%)
41–50 per year	0 (0%)	0 (0%)	0 (0%)	0 (0%)	0 (0%)
> 50 per year	0 (0%)	0 (0%)	1/1 (100%)	0 (0%)	0 (0%)

For the therapeutic approach to an asymptomatic pilonidal sinus, the most widespread strategy was the non-operative approach, watch and wait until symptoms arise, selected 17 times (44%). Operative management is also frequently chosen with a total of 14 votes (36%). Within the other alternatives, regular shaving was recommended four times (10%), laser hair removal three times (8%), and only one of the institutions stated that they do not suggest any treatment, not even a regular follow-up.

Concerning the treatment of a 6-month-old boy presenting with a deep sacral dimple with visible floor, the preferred option was watch and wait with 23 responses (59%). Nine institutions (23%) felt that no follow-up or treatment is needed, but seven of the respondents (18%) recommend prophylactic surgery. The results are provided stratified by surgical caseloads (Table 5).

**Table 5:** Approach to a six months old child with a deep sacral dimple

Caseload	No follow-up	Watch and wait	Prophylactic operation
< 5 per year	3/7 (14%)	3/7 (43%)	1/7 (14%)
5–10 per year	2/7 (29%)	4/7 (57%)	2/7 (29%)
11–20 per year	4/18 (22%)	11/18 (61%)	3/18 (17%)
21–30 per year	1/5 (20%)	3/5 (60%)	1/5 (20%)
31–40 per year	1/1 (100%)	0 (0%)	0 (0%)
41–50 per year	0 (0%)	0 (0%)	0 (0%)
> 50 per year	0 (0%)	1/1 (100%)	0 (0%)

Most institutions (30/38; 79%) stated that pilonidal sinus disease is common and this matches their experience in patient care, while three institutions (8%) feel it is a common problem and read or heard about it. However, five institutions (13%) classified pilonidal sinus as a rare problem and one respondent did not provide an answer to this question.

## Discussion

The incidence of pilonidal sinus disease is on the rise for adults [1, 2] as well as children and adolescents [3, 4]. Pilonidal sinus disease in the latter has some specific peculiarities with regard to temporal recurrence dynamics and increased recurrence rates [6–9]. These differences suggested that pilonidal sinus disease in minors might be different to those in adults, although the causative factors remain unknown. For pilonidal sinus disease in adults, several studies examining treatment realities in different countries are available demonstrating substantial differences in the surgical approach towards the disease [13, 15–18, 20]. Contrary to adults, only one from Turkey is available for paediatric pilonidal sinus disease [21]. We therefore examined the treatment reality of paediatric pilonidal sinus disease by German paediatric surgeons, representing a country with decentralised paediatric surgical care [22].

The recall rate of our study was 40%, which is more than doubled compared to the only other paediatric survey study by *Gurbanov* et al. [21] and within the range of surveys in the field of adult pilonidal sinus disease, which varied between 23% [16] and 55% [20]. In addition, the return rate is quite similar to the 38% return rate of German general surgery departments [13], indicating a similar level of representativeness. It might be even slightly higher as we were able to include respondents working outside hospitals in their own paediatric surgical doctor’s office, but could provide the full spectrum of procedures due to having associated hospital beds for inpatient care. This is of relevance, because Germany uses two largely separated healthcare sectors in parallel, one for inpatients and one for outpatients. If a study only covers one of them, this might impair the representativeness of the obtained results [25, 26]. However, excluding paediatric surgery offices without inpatient beds is also a limitation, because the majority of surgeries do not have inpatient beds and the spectrum of treatment could be different for office-based procedures only. Likewise, the recall rate itself, although within limits of other studies, could still be a limitation, because non-participating institutions might have different approaches. This would then result in a selection bias, because institutions favouring traditional approaches could be overrepresented in our surveyed sample.

The distribution of case numbers per year is similar to other surveys from general surgeons [15, 17, 20]. A notable exception is the study from Norway, which indicated smaller numbers of operations for pilonidal sinus disease per year for the vast majority of respondents [16]. The annual case distribution is however comparable to the study in Turkish paediatric surgeons, although the number of paediatric surgeons with a comparatively increased caseload was higher there [21]. This might be explained by a higher prevalence of pilonidal sinus disease in school children than in Germany [27], which would mirror the higher prevalence in adults [28, 29].

Substantial differences in the approach to patients with pilonidal sinus disease could be found in terms of the intraoperative use of blue dyes. Some 70% of general surgeons in Germany use blue dyes intraoperatively [13]. For comparison, 86% of Austrian general surgeons and even 90% in Switzerland report the use of blue dyes [18]. In our study, 62% of paediatric surgeons do not use them. Despite this striking difference in day-to-day practice, the non-usage is similar to the 72% of respondents that state not using a dye intraoperatively in a local Australian survey of general surgeons [30]. Notably, many surveys did not address this item [15–17, 20, 21, 31]. On a national level, the guideline recommends it use [32], but is not applicable to children and adolescents. The literature is divided on the issue whether the intraoperative use of blue-dyes decreases recurrence rates [30, 33–37]. However, with the exception of one prospective-randomised study [34], these are all retrospective studies with consecutively limited quality of evidence. A relevant limitation of our study with regard to blue dyes is that blue dyes are used to mark the system of sinuses for excisional procedures. Therefore, they would not be used for non-excisional procedures and our survey did not sufficiently delineate between these different approaches and might thus have caused distorted results.

Another item drafted to investigate guideline or recommendation adherence was the treatment of an acute pilonidal sinus with abscess formation: The national German guideline [32] recommends to wait until the inflammation has resolved after incision and drainage of the abscess, so do paediatric treatment recommendations [11, 38]. Despite the guideline recommendations, the majority of German general surgeons, 61%, prefer a one-stop-shop approach combining abscess incision and definitive excision of the areas affected by pilonidal sinus disease, which is a rarity in an international survey [31]. Much less common, only 1/3 of respondents stick to the two-step approach, in our study. However, an earlier version of the guideline suggested that in cases of limited disease, it might be suitable to perform a one-stage approach with pilonidal sinus excision at the time of abscess incision [39], but this recommendation had been revised in the second version [32]. Doing so offers the opportunity to spare the affect child or adolescent the excisional surgery if the pilonidal sinus disease had resolved on its own after incision and drainage, which is not uncommon [11, 12, 40].

The vast majority of surveyed German paediatric surgeons preferred traditional surgical approaches to pilonidal sinus disease — excision and wound closure by secondary intention or excision and vacuum-assisted closure. Contrary to these findings, Turkish paediatric surgeons favoured excision with primary midline closure before excision and wound closure by secondary intention [21]. The minimally invasive phenol treatment frequently used in Turkey indicates that at least some minimally invasive treatment options are implemented into day-to-day practice. This might be corroborated by our study, which used pit-picking in similar frequencies. German general surgeons also preferred traditional excisional techniques [13], which was also preferred by a majority of respondents from the Netherlands [15] and the UK [20]. General surgeons from Australia preferred an off-midline closure following excision of a pilonidal sinus [30]. This finding is in contrast to the recent literature, in which minimally invasive trephination is the dominant treatment approach [41–43]. Other frequently employed techniques are pit-picking [44], endoscopic [45] or laser therapy, and flap surgery [44, 46, 47]. Similar discrepancies are common and had been observed, for example, in paediatric inguinal hernia repair, in which the literature revolves around laparoscopic repair [48], but the treatment reality still is open herniotomy [49, 50].

While minimally invasive treatments are favoured in other countries [42, 43, 51–57], these approaches are seldom used by German paediatric surgeons. It has previously, although in a different healthcare system and with regard to the lack of the use of flap procedures in adults, been assumed that a lack training would be the causative factor for non-implementation of newer techniques into everyday care [58]. It might be tempting to speculate on a lack of training, given the increasing numbers of paediatric surgeons in Germany [59], but this is unlikely: Our survey showed that in the majority of institutions, the operations were performed by consultants or even the head of department. Moreover, the issue of training in paediatric surgery does not pertain to more complex flap procedures, but common surgical techniques for which detailed technical descriptions are available in the literature [52, 53]. A more apt explanation could be habit: It has been the common approach in the own training and the subsequent formative years, it is also the common approach in general surgery [13], and what colleagues at nearby institutions also likely practice.

Another aspect that might indirectly influence treatment decision is the structure of German hospital reimbursement. It is well described that outpatient care of pilonidal sinus disease results in a reimbursement that is only 1/3 of inpatient care reimbursement [60]. This might implicitly favour procedures that result in the necessity of an inpatient-treatment. This might explain the frequencies of negative-pressure wound therapy found in our survey as this is inevitably linked to inpatient care. With regard to the almost absence of endoscopic procedures in our study, this could be linked to the necessity of additional instruments and higher costs associated with the procedure. Hospital reimbursement in Germany is via a flat charge calculated from diagnoses and procedures, but if there is no specific code associated with the higher costs, these will have to be covered by the hospital. This has been already described for robotic surgery, whose implementation is hampered by the higher costs that have to be covered by the hospital, because they are not reimbursed [61].

Interestingly, general surgeons in the UK reported their own recurrence rates to vary between < 5% for 14% and up 30% for 29% [20], which is rather self-critical compared to only 13% that reported a recurrence rate above 15% in an international survey [31]. In our study, those respondents with lower caseloads reported a lower frequency of recurrent cases than those with higher caseloads. This might indicate a non-directional form of centralisation for recurrences, in the form of referral for recurrent disease, which is itself associated to a higher frequency of recurrences [62, 63]. Contrasting this with other results from the literature is difficult, as this item has often not been assessed elsewhere [13, 15–18, 21]. Support for this interpretation might be gained from studies that assessed preceding surgeries for pilonidal sinus disease among their cohort like 23% of those who underwent a Bascom flap [47] up to 41% of patients in a dedicated pilonidal sinus clinic [64]. Moreover, some of the recurrences might first manifest after patients have been transitioned to adult general surgery. Consequently, the perceived recurrence rate in day-to-day practice might be too low, because paediatric surgeons would not see them again when they reached adulthood.

The case vignettes, which are a common instrument in such studies to assess treatment approaches [17, 19, 20], revealed that a substantial part of respondents would still recommend prophylactic surgery although the national guideline strongly opposed such an approach since its inception [65]. Even the nineteenth century literature had already stated that a deep sacral dimple would be clinically irrelevant and not warrant surgical intervention [66, 67]. Likewise, more than a third of institutions recommended surgery for an asymptomatic pilonidal sinus disease, although the national guideline also opposed it, because it would not result in advantageous course of disease compared to watch-and-wait [32, 39, 68], which is the preferred approach to asymptomatic disease of almost half of the institutions.

We opted not to include inferential analyses in our study. First of all, during the design phase of the study, we had no knowledge on the treatment distribution of paediatric pilonidal sinus among paediatric surgical units in Germany. Therefore, it seemed possible that many departments would not treat pilonidal sinus disease in juveniles and the affected children and in particular adolescents could be treated by general surgeons. Paediatric surgery in Germany is highly decentralised [22] and the spectrum of treated paediatric surgical diseases varies substantially between departments [69]. Likewise, we had no prior knowledge on expected caseloads and recurrence rates, as this was among the reasons to conduct this survey. We therefore felt that substantial bias might be introduced in our analyses, which would then be hypothesis-generating at best. Another issue that precluded inferential analyses was that we did not ask for exact numbers of recurrence rates or treated patients per year, but preferred to stick to ranges for both numbers. We hoped that this might result in higher participation rates, because exact numbers for the department wouldn’t have to be looked up by the respondents. If exact numbers were to be provided, this could have been not only one of the past twelve months or the past year, due to the substantial variability per year. Especially if only low numbers of cases were treated at the respective centre, which made a range more convenient. Moreover, this was one of the precautions to preserve anonymity of the participating institutions. In Germany, all hospitals have to provide exact case numbers per year for quality control (*Strukturierter Qualitätsbericht*), which are publicly available if the number is at least four per diagnosis or procedure. Assessing a range would provide a similar level of information, although with less precision, but ensure anonymity. However, a range might introduce additional bias if the hospitals would potentially cluster at the lower or the upper limit of the range. This would have turned potential inferential analysis into being probably misleading, which we felt not appropriate.

A limitation of our study is that we did not ask for details of postoperative care and postoperative recommendations. At the time of the study design, there were no consistent postoperative recommendations in place [32] or even reliable data available to guide postoperative care. The trial on postoperative laser epilation that had been underway since 2018 [70], but was not completed then. Its results now show a relevant treatment effect in the reduction of postoperative recurrences of pilonidal sinus disease in children and adolescents if laser epilation was performed [71]. Although laser epilation is not equally beneficial for all patient groups [72], it would have been informative to know how many paediatric surgeons had already included this intervention in their daily practice. Another limitation might be that limiting the survey to paediatric surgeons might not have captured all treatment approaches towards children and adolescents. They might also be treated by general surgeons, which can be exemplified by age limitations imposed on a relevant number of paediatric surgical departments [69]. However, it is not very likely that those general surgeons treating children and adolescents would use different approaches than in adults. This might be assumed from the relatively homogenous treatment approach revealed by the survey of German general surgery departments on the treatment of pilonidal sinus disease [13]. Besides that, we aimed to investigate how paediatric surgeons would treat pilonidal sinus disease and not how children and adolescents in general would be treated in Germany. The next limitation by design is that we did not investigate the approach on an individual surgeon level, as many others did [15–17, 20, 21, 30, 31], but on a departmental level. Consequently, the individual paediatric surgeon’s level of training, experience, and perceptions towards treatment, failure, and disease is beyond the scope of our study, as this could not be expressed on the level of departments. Likewise, the recurrence rate is also aggregated on a departmental level based on the experiences of the department, but not assessed in a structured way or follow-up. Consequently, it might be different to the responses as these recurrence rates do not represent outcome data. This could have given rise to recall and response bias.

Taken together, German paediatric surgeons currently prefer traditional approaches to the surgical treatment of paediatric pilonidal sinus disease. In case of recurrent disease, the majority of respondents will use their initial technique again. Neither the adult-based national guideline recommendations nor those of proposed paediatric treatment algorithms have been widely introduced in day-to-day practice of paediatric surgeons in Germany.

## Supplementary Information

Below is the link to the electronic supplementary material.

Supplementary Information 1. Translated version of the survey questionnaire in the English language (DOCX 16.9 KB).

Supplementary Information 2. Original German version of the survey questionnaire (DOCX 18.2 KB).

## Data Availability

All data supporting the findings of this study are available within the paper and its supplementary information.
