# Supplementary material for: Practice patterns of paediatric surgeons on treating pilonidal sinus disease — a national survey study
Source: Int J Colorectal Dis. 2025 Jul 28;40(1):165. doi: 10.1007/s00384-025-04959-x (PMC12304014; doi:10.1007/s00384-025-04959-x)
Supplement: Supplementary file 1 — Supplementary Information 1. Translated version of the survey questionnaire in the English language (DOCX 16.9 KB). [file 384_2025_4959_MOESM1_ESM.docx]

**Survey on the management of paediatric pilonidal sinus disease in Germany – English version**

**1.**     **How many Sinus Pilonidalis operations do you perform annually in your clinic/practice?**

☐ 0

☐ < 5

☐ 5 -10

☐ 11 - 20

☐ 21 - 30

☐ 31 - 40

☐ 41 - 50

☐ > 50

**2.**     **How many of the operations are typically performed in case of recurrence?**

☐ < 10%

☐ 11-20%

☐ 21-30%

☐ > 30%

**3.**     **At what stage of training are the surgeons typically in your practice/clinic? (Multiple choice):**

☐ Resident

☐ Attending Physician

☐ Senior Physician/Consultant

☐ Chief Physician

**4.**     **Which procedure do you typically use in your clinic/practice? (Multiple choice)**

☐ Primary closure in the midline

☐ Primary closure off the midline (asymmetric or paramedian)

☐ Open procedure with secondary wound healing

☐ Open procedure with VAC-Therapy

☐ Minimally invasive pit-picking

☐ Minimally invasive sinusectomy

☐ Karydakis or Bascom flap surgery

☐ Limberg or Dufourment flap surgery

☐ Other procedure, namely: _______________________

**5.**     **Do you change the surgical procedure in your clinic/practice after a recurrence?**

☐ No

☐ Yes, to primary closure in the midline

☐ Yes, to primary closure off the midline (asymmetric or paramedian)

☐ Yes, to open procedure with secondary wound healing

☐ Yes, to open procedure with VAC therapy

☐ Yes, to minimally invasive pit-picking

☐ Yes, to minimally invasive sinusectomy

☐ Yes, to Karydakis or Bascom flap surgery

☐ Yes, to Limberg or Dufourment flap surgery

☐ Yes, to another procedure, namely: _______________________

**6.**     **Do you typically use a dye intraoperatively, such as Methylene Blue, Toluidine Blue, or Patent Blue?**

☐ Yes

☐ No

**7.**     **Who typically provides the follow-up care after surgery for a pilonidal sinus?**

☐ Operating clinic/practice

☐ Private surgeon

☐ Private pediatrician/general practitioner

**8.**     **How is an acutely abscessed pilonidal sinus typically treated in your clinic/practice?**

☐ Single-stage, abscess drainage and definitive treatment in one procedure

☐ Two-stage, abscess drainage and definitive treatment within 4 weeks

☐ Two-stage, abscess drainage and definitive treatment with more than 4 weeks’ interval

**9.**     **How is an asymptomatic pilonidal sinus typically treated in your clinic/practice? (Multiple choice)**

☐ Conservative management

☐ Regular shaving

☐ Laser hair removal

☐ Surgical intervention

☐ Other measure, namely: _______________________

**10.**  **Scenario 1: A 6-month-old boy presents with a deep sacral dimple suspected of being an asymptomatic, congenital pilonidal sinus. What is your usual approach?**

☐ Conservative treatment

☐ Prophylactic surgery

☐ No follow-up or treatment needed

**11.**  **Scenario 2: A 12-year-old girl presents with an acutely abscessed pilonidal sinus. Which procedure would you choose for definitive surgical treatment 6 weeks after abscess drainage?**

☐ Primary closure in the midline

☐ Primary closure off the midline

☐ Open procedure

☐ Minimally invasive procedure

☐ Flap surgery

**12.**  **Scenario 3: A 17-year-old boy presents with an acutely abscessed pilonidal sinus. Which procedure would you choose for definitive surgical treatment 4 weeks after abscess drainage?**

☐ Primary closure in the midline

☐ Primary closure off the midline

☐ Open procedure

☐ Minimally invasive procedure

☐ Flap surgery

**13.**  **Variant Scenario 3: Would your choice of procedure be influenced if the boy reports that he will be staying for a year in a developing country?**

☐ No

☐ Yes, now primary closure in the midline

☐ Yes, now primary closure off the midline

☐ Yes, now open procedure

☐ Yes, now minimally invasive procedure

☐ Yes, now flap surgery

**14.**  **Do you believe that pilonidal sinus is a common and increasingly prevalent problem in Germany?**

☐ No, it is a rare problem

☐ Yes, it is common, I read/heard about it

☐ Yes, it is common, and this matches my experience in patient care
