# Supplementary material for: Practice patterns of paediatric surgeons on treating pilonidal sinus disease — a national survey study
Source: Int J Colorectal Dis. 2025 Jul 28;40(1):165. doi: 10.1007/s00384-025-04959-x (PMC12304014; doi:10.1007/s00384-025-04959-x)
Supplement: Supplementary file 2 — Supplementary Information 2. Original German version of the survey questionnaire (DOCX 18.2 KB). [file 384_2025_4959_MOESM2_ESM.docx]

**Fragenkatalog für die Umfrage zum Sinus pilonidalis**

1. Wie viele Operationen eines Sinus pilonidalis führen Sie in ihrer Klinik/Praxis üblicherweise durch?

☐ Keine

☐ 1-5 pro Jahr

☐ 6-10 pro Jahr

☐ 11-20 pro Jahr

☐ 21-30 pro Jahr

☐ 31-40 pro Jahr

☐ 41-50 pro Jahr

☐ >50 pro Jahr

2. Wie viele dieser Operationen finden in etwa bei Rezidiven statt?

☐ <10%

☐ 11-20%

☐ 21-30%

☐ >30%

3. In welchem Ausbildungsstand befinden sich üblicherweise die Operateur:innen in ihrer Praxis/Klinik? [Mehrfachauswahl]

☐ Weiterbildungsassistent:in

☐ Fachärzt:in

☐ Oberärzt:in

☐ Chefärzt:in/Praxisinhaber:in

4. Welches Verfahren wenden Sie in ihrer Klinik/Praxis üblicherweise an? [Mehrfachauswahl]

☐ Primärverschluss in der Mittellinie

☐ Primärverschluss abseits der Mittellinie (asymmetrisch oder paramedian)

☐ Offenes Verfahren mit sekundärer Wundheilung

☐ Offenes Verfahren mit VAC-Anlage

☐ Minimalinvasives Pit-Picking

☐ Minimalinvasive Sinusektomie

☐ Karydakis- oder Bascomlappenplastik

☐ Limberg- oder Dufourmentellappenplastik

☐ Anderes Verfahren, und zwar: _______________________

5. Wechseln Sie in ihrer Klinik/Praxis nach einem Rezidiv das operative Verfahren?

☐ Nein

☐ Ja, auf Primärverschluss in der Mittellinie

☐ Ja, auf Primärverschluss abseits der Mittellinie (asymmetrisch oder paramedian)

☐ Ja, auf offenes Verfahren mit sekundärer Wundheilung

☐ Ja, auf offenes Verfahren mit VAC-Anlage

☐ Ja, auf minimalinvasives Pit-Picking

☐ Ja, auf minimalinvasive Sinusektomie

☐ Ja, auf Karydakis- oder Bascomlappenplastik

☐ Ja, auf Limberg- oder Dufourmentellappenplastik

☐ Ja, auf anderes Verfahren, und zwar: _______________________

6. Wenden Sie üblicherweise intraoperativ einen Farbstoff an wie beispielsweise Methylen-/Toluidin-/Patentblau?

☐ Ja

☐ Nein

7. Durch wen erfolgt üblicherweise die Nachsorge nach der Operation eines Sinus pilonidalis?

☐ Operierende Klinik/Praxis

☐ Niedergelassener Chirurg:in

☐ Niedergelassener Kinder-/Hausärzt:in

8. Wie wird in ihrer Klinik/Praxis ein akut abszedierender Sinus pilonidalis üblicherweise behandelt?

☐ Einzeitig, Abszesspaltung und definitive Versorgung in einer Prozedur

☐ Zweizeitig, Abszessspaltung und definitive Versorgung innerhalb von 4 Wochen

☐ Zweizeitig, Abszesspaltung und definitive Versorgung mit mehr als 4 Wochen Abstand

9. Wie wird in ihrer Klinik/Praxis ein asymptomatischer Sinus pilonidalis üblicherweise behandelt? [Mehrfachauswahl]

☐ Abwartend

☐ Regelmäßige Rasur

☐ Laserdepilation

☐ Operativ

☐ Andere Maßnahme, und zwar: _______________________

10. Szenario 1: Ein 6 Monate alter Junge wird Ihnen mit einem tiefen Sakralgrübchen unter dem Verdacht auf einen asymptomatischen, kongenitalen Sinus pilonidalis vorgestellt. Welches Vorgehen ist bei Ihnen üblich?

☐ Abwartende Behandlung

☐ Prophylaktische Operation

☐ Keine Kontrolle und keine Maßnahmen erforderlich

11. Szenario 2: Ein 12-jähriges Mädchen wird mit einem akut abszedierenden Sinus pilonidalis vorgestellt. Welches Verfahren würden Sie 6 Wochen nach Entdeckelung des Abszesses für die definitive operative Versorgung wählen?

☐ Primärverschluss in der Mittellinie

☐ Primärverschluss abseits der Mittellinie

☐ Offenes Verfahren

☐ Minimalinvasives Verfahren

☐ Lappenplastik

12. Szenario 3: Ein 17-jähriger Junge wird mit einem akut abszedierenden Sinus pilonidalis vorgestellt. Welches Verfahren würden Sie 4 Wochen nach Entdeckelung des Abszesses für die definitive operative Versorgung wählen?

☐ Primärverschluss in der Mittellinie

☐ Primärverschluss abseits der Mittellinie

☐ Offenes Verfahren

☐ Minimalinvasives Verfahren

☐ Lappenplastik

13. Abwandlung Szenario 3: Würde es Ihre Verfahrenswahl beeinflussen, wenn der Junge Ihnen berichtet, dass er sich in absehbarer Zeit für ein Jahr in einem wenig entwickelten Land aufhalten würde?

☐ Nein

☐ Ja, nunmehr Primärverschluss in der Mittellinie

☐ Ja, nunmehr Primärverschluss abseits der Mittellinie

☐ Ja, nunmehr offenes Verfahren

☐ Ja, nunmehr minimalinvasives Verfahren

☐ Ja, nunmehr Lappenplastik

14. Sind Sie der Ansicht, dass es sich beim Sinus pilonidalis um ein häufiges und weiter zunehmendes Problem in Deutschland handelt?

☐ Nein, es ist ein seltenes Problem.

☐ Ja, es ist häufig, ich las/hörte davon

☐ Ja, es ist häufig und dies entspricht auch meiner Wahrnehmung aus der Patient:innenversorgung
